# From Fiber Bundles to Architected Membranes: Triply Periodic Minimal Surface Architectures for Biohybrid Artificial Lungs

**DOI:** 10.1002/adma.74361

**Published:** 2026-08-03

**Authors:** Michael Pflaum, Kai P. Barbian, Florian Neuhaus, Gerrit Sitarz, Carolin Nölke, Sebastian V. Jansen, John Linkhorst, Lukas T. Hirschwald, Sebastian Brosch, Christian Certa, Ulrich Steinseifer, Matthias Wessling, Jutta Arens, Arjang Ruhparwar, Bettina Wiegmann

**Affiliations:** ^1^ Department of Cardiothoracic, Transplantation, and Vascular Surgery Hannover Medical School Hannover Germany; ^2^ Lower Saxony Centre for Biomedical Engineering Implant Research and Development (NIFE) Hannover Germany; ^3^ German Center for Lung Research (DZL) Breath Hannover Germany; ^4^ Department of Cardiovascular Engineering Institute of Applied Medical Engineering Medical Faculty RWTH Aachen University Aachen Germany; ^5^ AVT.CVT ‐ Chair of Chemical Process Engineering RWTH Aachen University Aachen Germany; ^6^ Process Engineering of Electrochemical Systems Technical University Darmstadt Darmstadt Germany; ^7^ Member of the DFG Priority Program SPP2014: Towards an Implantable Lung Aachen Germany; ^8^ DWI ‐ Leibniz‐Institute for Interactive Materials RWTH Aachen University Aachen Germany; ^9^ Engineering Organ Support Technologies Group Department of Biomechanical Engineering University of Twente Enschede Netherlands

**Keywords:** additive manufacturing, architected membranes, artificial lung, biohybrid lung, endothelialization, triply periodic minimal surfaces

## Abstract

Artificial lung systems rely almost exclusively on hollow fiber membrane (HFM) bundles, where gas exchange is constrained by heterogeneous flow distribution and thrombogenic blood–material interfaces. Here, we introduce an architecture‐driven design framework for artificial lungs based on additively manufactured triply periodic minimal surface (TPMS) membranes. In contrast to discrete fiber bundles, TPMS membranes form continuous three‐dimensional architectures that simultaneously regulate perfusion pathways, diffusion interfaces, and blood–material interactions. Computational fluid dynamics and multiphysics transport simulations reveal that membrane architecture governs gas exchange through coupled effects of membrane thickness, unit cell size, and three‐dimensional flow topology. Optimized TPMS architectures achieved on average up to ∼88% higher oxygen transfer rates across the investigated flow regime compared to conventional HFM while enabling substantially more homogeneous flow fields and reduced stagnation zones. Experimental screening identifies polydimethylsiloxane‐based printable elastomers compatible with thin gas‐permeable membranes and endothelial functionalization. The biohybrid endothelial interface mitigates thrombogenic interactions, while maintaining gas transport. Computed tomography–derived implant geometries demonstrate the feasibility of translating architected membrane systems into anatomically integrated artificial lungs. Together, these results establish a new design paradigm for artificial lungs, in which membrane architecture becomes the primary determinant of gas transport, flow distribution, and hemocompatibility.

## Graphical abstract

Additively manufactured triply periodic minimal surface (TPMS) membranes offer an architecture‐driven alternative to hollow fiber bundles in artificial lungs. Multiphysics simulations and endothelialized prototypes show that the 3D‐printable membrane architecture improves gas exchange, blood flow distribution, and hemocompatibility, enabling homogeneous perfusion and sustained gas transfer. These findings support the development of patient‐tailored implantable TPMS oxygenators for long‐term lung support.

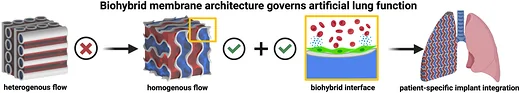

**Abbreviations**
3D: three‐dimensional
BCECF‐AM: 2′,7′‐Bis‐(2‐Carboxyethyl)‐5‐(and‐6)‐Carboxyfluorescein, Acetoxymethyl ester
BHL: biohybrid lung
BSA: bovine serum albumin
Calcein‐AM: calcein‐acetoxymethyl ester
cDNA: complementary deoxyribonucleic acid
CFD: computational fluid dynamics
CT: computed tomography
Ct: cycle threshold
DA: dynamic accumulation
DLP: digital light processing
DPBS: Dulbecco's phosphate‐buffered saline
ECMO: extracorporeal membrane oxygenation
ECs: endothelial cells
EDTA: ethylenediaminetetraacetic acid
EDX: energy‐dispersive x‐ray
EGM‐2: endothelial growth medium‐2
EML: endothelial monolayer
FN: fibronectin
HFM: hollow fiber membrane
IPA: isopropanol
MAP‐PDMS: methylsiloxane‐based PDMS
OR: oxygen‐rich chamber
OTR: oxygen transmission rate
PBS: phosphate‐buffered saline
PCR: polymerase chain reaction
PDMS: polydimethylsiloxane
PEG: polyethylene glycol
PMP: poly(4‐methyl‐1‐pentene)
RNA: ribonucleic acid
SD: standard deviation
SEM: scanning electron microscopy
SLS: selective laser sintering
STL: standard tessellation language
SWD: Schwarz Diamond
TCP: tissue culture plastic
TNFα: tumor necrosis factor alpha
TPMS: triply periodic minimal surfaces
TPU: thermoplastic polyurethane
Tris: tris(hydroxymethyl)aminomethane
VE‐cadherin: vascular endothelial‐cadherin
WST: water‐soluble tetrazolium
xPDMS: methacrylate‐functionalized crosslinkable PDMS

## 1 Introduction

Global burden of end‐stage lung diseases continues to rise, driven by demographic change and increasing prevalence of chronic respiratory disorders such as chronic obstructive pulmonary disease (COPD) [1]. As lung transplantation remains the only curative therapy, declining donor organ availability has intensified a structural mismatch between available grafts and clinical demand [2, 3]. Extracorporeal membrane oxygenation (ECMO) therefore remains indispensable as temporary life support. Despite substantial technological refinement and expanded clinical implementation [4, 5, 6, 7, 8, 9], ECMO continues to be associated with considerable morbidity and mortality [7, 10, 11, 12], mandatory systemic anticoagulation [2], and confinement to intensive care settings. As a consequence, current oxygenator systems are limited to short‐term support and have not translated into durable, wearable lung replacement or final‐destination therapy, in contrast to left ventricular assist devices in advanced heart failure [13]. In clinically established ECMO oxygenators, blood is guided along the exterior of densely packed hollow fiber membranes (HFM) composed of poly(4‐methyl‐1‐pentene) (PMP), while oxygen‐rich sweep gas flows within the fiber lumina. Gas exchange occurs by diffusion across the membrane wall along established partial pressure gradients. To achieve adequate oxygenation in adults, membrane surface areas exceeding 1.8 m^2^ are required [14], thereby exposing circulating blood to extensive artificial interfaces. By virtue of its topology, the fiber‐bundle architecture links gas transfer performance to thrombogenic risk. Laminar or even creeping flow along individual fibers promotes boundary layer formation, reducing effective diffusion gradients and thus limiting gas exchange efficiency [15, 16]. Simultaneously, the densely packed bundle geometry generates heterogeneous flow fields characterized by regions of elevated shear stress at inlet and outlet zones [17] and areas of low velocity or stagnation within the bundle core [18]. Both extremes, high shear and low‐flow conditions, promote hemolysis, platelet activation, and thrombus formation. In parallel, unavoidable adsorption of plasma proteins and platelets onto artificial surfaces initiates coagulation cascades [19, 20], which cannot be fully prevented by heparin coatings or systemic anticoagulation and ultimately result in severe hemorrhagic or thromboembolic complications [21]. Consequently, thrombus formation with progressive loss of gas exchange capacity remains the most frequent complication of ECMO therapy [22].

Several strategies have been proposed to overcome these limitations, including advanced surface modifications, novel membrane materials, microfluidic oxygenators, and computationally optimized oxygenator designs [21, 23, 24, 25, 26, 27]. Within this broad landscape, the present study focuses on two complementary concepts. The first centers on the biohybrid lung (BHL) concept, which seeks to restore physiological hemocompatibility by covering blood‐contacting surfaces with a viable endothelial monolayer (EML) [28, 29, 30, 31, 32]. Endothelial cells actively regulate coagulation and inflammation via nitric oxide production [33] and expression of anticoagulant molecules such as thrombomodulin [34]. Stable and confluent endothelialization has been demonstrated on PMP‐based HFM and oxygenator housings [30, 31, 32, 35, 36, 37], maintaining integrity under flow while significantly reducing platelet adhesion without impairing gas transfer [18, 31, 38, 39, 40]. The second concept introduces a fundamentally different membrane architecture based on triply periodic minimal surfaces (TPMS) [41, 42]. TPMS are mathematically defined, continuous three‐dimensional geometries with zero mean curvature that partition space into two interwoven but non‐communicating compartments. In contrast to discrete fiber bundles, TPMS enable continuous flow channels with tunable curvature and length scales, facilitating more homogeneous velocity fields and improved mass transport. Computational fluid dynamics and experimental analyses suggest that, depending on the material, TPMS‐based oxygenators can achieve significantly enhanced gas exchange performance [42, 43, 44, 45, 46]. Moreover, their digital construct enables direct integration of computed tomography (CT)‐derived anatomical data, opening the possibility of volumetrically integrated, patient‐specific 3D lung replacements. Additive manufacturing is indispensable for realizing such complex individual architectures [47]. However, geometric freedom alone does not ensure functional gas exchange. Translational implementation of TPMS‐based lung constructs requires materials that combine selective gas permeability, liquid‐tight separation of compartments, mechanical stability at micrometer‐scale wall thickness, additive manufacturing compatibility, and biological hemocompatibility permitting endothelialization. Non‐porous polydimethylsiloxane (PDMS) represents a promising candidate, characterized by high O_2_ and CO_2_ permeability, mechanical compliance, and established biomedical applicability, including microfluidic artificial lung systems [23, 47]. Building upon these concepts, we propose a design paradigm shift from fiber‐based membrane bundles toward architected, additively manufactured minimal surface membranes. Rather than treating gas transfer, flow dynamics, and hemocompatibility as independent optimization problems, this approach integrates them through the underlying membrane architecture. In this framework, membrane architecture itself becomes a primary design variable that simultaneously governs gas transport, flow distribution, and hemocompatibility in artificial lung systems. Using computational fluid dynamics‐guided optimization of channel size and membrane thickness, CT‐based anatomical adaptation, systematic material screening for gas transfer and hemocompatibility, and three‐dimensional (3D) endothelialization of TPMS constructs, we establish a translational in vitro validation framework for a 3D BHL. This integrated strategy reframes oxygenator development from incremental component refinement to geometry‐driven and biologically integrated system design.

## 2 Materials and Methods

### 2.1 Architecture Design and Flow Simulation

#### 2.1.1 TPMS Geometry and Membrane Architecture

Triply periodic minimal surfaces (TPMS) geometries were generated using the Schwarz Diamond topology. Periodic unit‐cell assemblies were created with unit cell sizes of 1 and 4 mm and membrane wall thicknesses of 10, 100, and 400 µm. For calculation of geometry‐specific surface areas, high‐resolution TPMS models consisting of 10 × 10 × 10 unit‐cell assemblies were generated and numerically evaluated to determine the corresponding volume‐specific surface area (m^2^ m^−^^3^) for each geometry configuration. Geometries of the corresponding fluid domain were generated using custom MATLAB code (MathWorks, USA) and exported as standard tessellation language (STL) surface mesh files for subsequent meshing and computational fluid dynamics (CFD) simulation. Meshing was performed in ANSYS ICEM CFD (ANSYS Inc., USA) for subsequent CFD simulation of multiphysics flow fields and gas transfer.

#### 2.1.2 CFD Simulation of Flow Fields and Velocity Distribution

CFD simulations were performed to compare flow characteristics within TPMS and HFM architectures. The computational domain consisted of 3 × 2 × 2 (X × Y × Z) unit cells for each geometry, analogous to previously described models [24]. A mesh convergence study was conducted to ensure numerical stability, resulting in discretizations of 32.6 million elements for TPMS geometries and 16.9 million elements for HFM geometries. Blood flow was modeled as a single‐phase incompressible fluid. To characterize flow homogeneity within the different architectures, velocity distributions and local flow fields were evaluated throughout the computational domain. Particular attention was given to the identification of stagnation regions, which were defined as areas with local flow velocities below 1 mm/s. These regions were identified by velocity thresholding to quantify potential flow stagnation zones. In addition, histograms of fluid volume fractions as a function of local flow velocity magnitude were generated to quantitatively compare velocity distributions between HFM and TPMS architectures. Velocity magnitude distributions were evaluated throughout the entire computational domain to assess flow homogeneity and the relative fraction of low‐velocity regions. The resulting velocity fields and streamline patterns were analyzed to compare the spatial distribution of flow within hollow fiber membrane (HFM) bundles and TPMS architectures. All simulations were performed using ANSYS CFX 2021 R1 (ANSYS Inc., USA).

### 2.2 Multiphysics Simulation of Gas Transfer

#### 2.2.1 Multiphysics Simulation of Oxygen and Carbon Dioxide Transfer

Gas transfer simulations were performed to evaluate architecture‐dependent oxygen and carbon dioxide transport within TPMS and HFM membrane configurations. O_2_ and CO_2_ transport were modeled using the validated approach described by Hormes, which incorporates a local effective diffusion coefficient within a single‐phase fluid model [48]. This model accounts for gas uptake into erythrocytes by considering the slope of the respective oxygen and carbon dioxide dissociation curves. For HFM simulations, membrane mass transfer resistance was neglected due to its minor contribution to overall transport resistance in this configuration [25]. In contrast, for dense TPMS membranes, mass transfer resistance across the membrane was included and calculated based on membrane thickness and material diffusivity. Reported diffusivity values for polydimethylsiloxane (PDMS) range from 5.2 × 10^−^^10^ to 7.88 × 10^−^^9^ m^2^ s^−^^1^ for O_2_ and 1.1 × 10^−^^9^ to 4.2 × 10^−^^9^ m^2^ s^−^^1^ for CO_2_ [49, 50, 51, 52, 53, 54, 55]. To account for variability in reported material properties, uncertainty quantification was performed by incorporating the median as well as the 25th and 75th percentile diffusivity values into the simulations [56]. Gas transport was simulated independently for each membrane configuration. Inlet partial pressures were set to 36 mmHg for O_2_ and 47 mmHg for CO_2_, in accordance with ISO 7199 [57]. Area‐specific gas transfer rates (j″) were evaluated for membrane thicknesses of 10, 100, and 400 µm to investigate the influence of membrane mass transfer resistance on overall gas transport. Simulations were conducted at physiologically relevant flow velocities of 20–45 mm s^−1^, corresponding to flow rates of approximately 60–140 mL min^−1^ within the modeled three‐dimensional lung construct.

### 2.3 Materials and Fabrication

#### 2.3.1 Additive Manufacturing Materials and Sample Preparation

Additive manufacturing‐compatible materials were screened for fabrication of TPMS‐based membrane structures combining gas permeability with liquid retention. PDMS, a dense polymer enabling diffusive gas transport [56], was investigated in digital light processing (DLP)‐printable formulations. Methacryloxypropyl methylsiloxane‐based PDMS (MAP‐PDMS) and methacrylate‐functionalized crosslinkable PDMS (xPDMS) were analyzed, differing in copolymer composition and photoinitiator content. In addition, the PDMS‐free resin PorePro, formed via photo‐induced phase separation resulting in a microporous polymer network, was evaluated as a DLP‐printable alternative. As a second additive manufacturing strategy, selective laser sintering (SLS) was employed to fabricate TPMS‐shaped scaffolds from thermoplastic polyurethane (TPU) powder (FlexaGrey, Sinterit, Poland). Due to their intrinsic porosity, SLS‐fabricated scaffolds were subsequently coated with a thin, liquid‐tight yet gas‐permeable PDMS layer on the blood‐facing surface. Accordingly, “FlexaGrey” refers to the uncoated porous TPU scaffold material, whereas “FlexaGrey + PDMS” denotes the same structure after application of the dense PDMS sealing layer. Disc‐shaped samples (20 mm diameter, 2 mm thickness) were fabricated from each material (Table 1) to fit standard 12‐well plates for in vitro testing. Pure PDMS discs were prepared by mixing Sylgard 184 base and curing agent (10:1 ratio; Dow Chemical, Germany), casting into Petri dishes, curing at 55°C, and punching to final dimensions. MAP‐PDMS [40], xPDMS (modified after Fleck et al. [58]), and PorePro resin (40 vol% monomer concentration) [59] were fabricated according to established protocols and printed via DLP with a layer thickness of 50 or 100 µm. For xPDMS fabrication, PDMS‐S ((7%–9% methacryloxypropyl‐methylsiloxane)‐dimethylsiloxane; Gelest Inc., USA) and PDMS‐E (methacryloxypropyl‐terminated polydimethylsiloxane; abcr GmbH, Germany) were blended in opaque containers to prevent premature photopolymerization. The resin formulation consisted of 80 wt% PDMS‐S and 20 wt% PDMS‐E supplemented with 0.6 wt% liquid BAPO (bis(2,4,6‐trimethylbenzoyl) octyl phosphine oxide; iGM Resins, Italy) and 3 wt% TPO‐L (ethyl(2,4,6‐trimethylbenzoyl)‐phenyl phosphinate; TCI Europe N.V., Belgium) as photoinitiators. Resins were homogenized using a speed mixer (Hauschild SpeedMixer DAC 600.2 CM 153, Germany) at 1000 rpm for 1 min, 2000 rpm for 1.5 min, and 500 rpm for 15 s, followed by storage in UV‐light‐protected containers. Prior to printing, resins were remixed under identical conditions to minimize photoinitiator sedimentation. MAP‐PDMS and PorePro were printed using a Photon Mono SE (Anycubic, China), whereas xPDMS was printed using a Sonic Mini 8K (Phrozen, Taiwan) operating at a wavelength range of 385–405 nm. For xPDMS printing, a layer thickness of 100 µm and an exposure time of 15 s were applied. Following fabrication, samples were immersed in ethanol and cleaned in an ultrasonic bath for 3 min. Samples were subsequently washed in water for at least 2 h to remove residual resin and ethanol from the printed structures. After washing, samples were post‐cured under UV‐light (405 nm) for 15 min to ensure complete photoinitiator conversion. FlexaGrey scaffolds were printed via SLS using a Lisa Pro system (Sinterit, Poland) with a layer thickness of 125 µm according to established protocols [60]. Heparin/albumin‐coated poly(4‐methyl‐1‐pentene) hollow fiber membranes (PMP‐HFM) sterilized with ethylene oxide (HA2 Medizintechnik GmbH, Germany) served as reference material.

**TABLE 1:** Selected materials and abbreviations. PMP—poly(4‐methyl‐1‐pentene); TIPS—thermally induced phase separation; PDMS—polydimethylsiloxane; MAP—methacryloxypropyl methylsiloxane; TPU—thermoplastic polyurethane; HEMA ‐ 2‐hydroxyethyl methacrylate; EGDMA—ethylene glycol dimethacrylate; PDMS‐E—methacryloxypropyl terminated polydimethylsiloxane; DLP—digital light processing; SLS—selective laser sintering.

Material	PMP	PDMS	FlexaGrey		PorePro		xPDMS	MAP‐PDMS
**Coating**	Heparin + Albumin	—	—	+ PDMS	—	+ PDMS	—	—
**Manufacturer**	3 M Membrana	Dow Chemical	Sinterit	Sinterit + self‐made	Self‐made	Self‐made	Self‐made	Self‐made
**Main constituent**	PMP	PDMS	TPU	TPU	HEMA	HEMA + EGDMA + PDMS	MAP‐PDMS	MAP‐PDMS
				+	+		+	
				PDMS	EGDMA		PDMS‐E	
**Manufacturing technique**	TIPS	chemical reaction	SLS	SLS + PDMS coating	DLP	DLP + PDMS coating	DLP	DLP

#### 2.3.2 PDMS Coating Strategy

Porous substrates (FlexaGrey and PorePro) were dip‐coated with PDMS to generate a thin, plasma‐tight barrier layer. FlexaGrey samples were oxygen plasma‐activated to increase surface hydrophilicity and thereby enable controlled pre‐wetting of the porous network with deionized water, ethanol, or isopropanol (IPA). Pre‐wetting was applied to modulate subsequent PDMS infiltration behavior and reduce capillary‐driven penetration into the macroporous structure, aiming to obtain a thinner and more surface‐confined PDMS coating layer. PorePro samples were washed repeatedly in IPA until no discoloration occurred, followed by an additional 5 min incubation in IPA. Samples were then dip‐coated in a diluted PDMS solution (85 vol% PDMS, 15 vol% dichloromethane). After 15 s immersion, excess solution was removed and samples were cured at 55°C for 24 h. All specimens underwent two sequential washing cycles in IPA and distilled water (15 min each, ultrasonic bath) and were dried for 24 h prior to further analysis.

### 2.4 Membrane Characterization and Gas Permeability Measurements

#### 2.4.1 Surface Morphology and Coating Thickness

The internal morphology of the printed materials and the thickness of the PDMS coating layers were analyzed by scanning electron microscopy (SEM) using a Crossbeam XB540 microscope (Zeiss, Germany). Samples with and without PDMS coating were cryo‐fractured after immersion in liquid nitrogen to obtain clean cross‐sectional surfaces. Fractured specimens were mounted with the cross‐sectional plane facing upward and sputter‐coated with a thin gold layer using a Polaron High Resolution Sputter Coater E5400 (Polaron Equipment Ltd., UK) prior to imaging. SEM images were acquired to assess internal morphology, coating uniformity, and PDMS infiltration into porous substrates. The mean PDMS coating thickness on FlexaGrey and PorePro substrates was determined using Fiji software (ImageJ 1.54p, NIH, USA). For each sample, nine independent measurements per SEM image were obtained and averaged to determine the mean coating thickness. Surface morphology and elemental composition were further examined using a tabletop SEM (Hitachi TM3030) equipped with an energy‐dispersive x‐ray (EDX) detector, enabling identification of PDMS coating distribution via silicon signals.

#### 2.4.2 Porosity Measurements

Porosity of the three‐dimensional (3D)‐printed FlexaGrey and PorePro disc‐shaped samples was characterized using a capillary flow porometer (IB‐FT Porolux 1000, Aptco Technologies NV, Belgium). Nitrogen flow through wetted membranes was recorded under stepwise increasing transmembrane pressure to determine pore opening behavior and pore size distribution. In addition, total pore volume was determined gravimetrically by comparing the mass of samples dried at 55°C for 24 h with samples fully saturated in isopropanol.

#### 2.4.3 Oxygen Permeability Measurements

Oxygen permeability was determined using a custom‐built dual‐chamber glass apparatus. Disc‐shaped samples (20 mm diameter) were mounted between an oxygen‐rich (OR) chamber and a dynamic accumulation (DA) chamber, each with a volume of 10 mL. The DA chamber was purged with 99.9% nitrogen for 20 s and subsequently sealed. Pure oxygen was continuously flushed through the OR chamber at atmospheric pressure. The increase in oxygen concentration within the DA chamber was recorded at 1 min intervals for 3 h at room temperature using a non‐invasive optical oxygen sensor (PreSens, Regensburg, Germany). The oxygen transmission rate (OTR) was calculated from three independent measurements according to a previously published method [61]. Sample thickness was measured using a digital caliper (Profi Scale Precise PS 7215, Burg Wächter, Germany). Oxygen permeability P was calculated using:

$$Permeability\;P=\frac{\left(OTR\;\times \;d\right)}{\Delta p}$$

where

- OTR = oxygen transmission rate (converted from mL m^−^^2^ day^−^^1^ to mol m^−^^2^ s^−^^1^)
- d = sample thickness (m)
- Δp = oxygen partial pressure difference (Pa).

### 2.5 In Vitro Biocompatibility and Hemocompatibility

#### 2.5.1 Endothelial Cell Culture

Primary human endothelial cells (ECs) were isolated from umbilical cord blood after informed consent and approval by the Institutional Review Board (IRB No. 1303‐2012), as previously described [30]. Cells were cultured on tissue culture plastic (TCP; Nunc Delta surface, Thermo Scientific, Germany) in endothelial growth medium (EGM‐2, Lonza, Germany) under standard cell culture conditions (37°C, 5% CO_2_). For routine maintenance, ECs were seeded at a density of 8 × 10^3^ cells cm^−^^2^ and passaged at 80%–90% confluence using enzymatic detachment with trypsin/ethylenediaminetetraacetic acid (EDTA) solution (Capricorn, Germany). Unless otherwise stated, ECs at passage 8 were used for all biocompatibility and endothelialization experiments.

#### 2.5.2 Extract Cytotoxicity Assay

Cytotoxicity of the printed and post‐processed materials was evaluated using a metabolic activity assay in accordance with DIN EN ISO 10993‐5 and ISO 10993‐12 [62, 63]. In contrast to the fibroblast cell lines recommended by the standard, primary human ECs were used to better reflect the physiological environment of blood‐contacting biomaterials. Material extracts were prepared by incubating samples in EGM‐2 medium at 37°C for 24 h. Extraction ratios were defined according to ISO 10993‐12. For non‐porous materials (PDMS‐coated FlexaGrey, PDMS‐coated PorePro, MAP‐PDMS, xPDMS, and PMP‐HA), a surface area‐to‐medium ratio of 3 cm^2^ mL^−1^ was applied. For porous materials (FlexaGrey and PorePro), a mass‐to‐medium ratio of 0.1 g mL^−1^ was used. Extract preparation was performed in quadruplicate. For cytotoxicity testing, ECs were seeded 24 h prior to exposure in 96‐well plates (Thermo Fisher, Germany) at a density of 5 × 10^3^ cells per well in 250 µL EGM‐2 medium. After medium removal, cells were incubated with the respective material extracts for 24 h. Cell metabolic activity was quantified using a water‐soluble tetrazolium (WST)‐8 assay (Promocell, Germany). Extracts were replaced with fresh EGM‐2 containing WST‐8 substrate (final dilution 1:11) and incubated for 4 h at 37°C. Absorbance was measured at 450 nm using a microplate reader (Synergy‐2, BioTek, Germany). Cell viability was expressed as percentage relative to ECs cultured in standard EGM‐2 medium (100% viability control). Materials were considered non‐cytotoxic when viability exceeded 80%, in accordance with ISO 10993‐5.

#### 2.5.3 Direct Contact Endothelialization Assay

Direct cell–material interaction was evaluated using a contact assay on the basis of ISO 10993‐5 recommendations. Disc‐shaped samples of the respective materials were placed into individual wells of 12‐well plates (Thermo Fisher Scientific, Germany) and seeded with 5 × 10^4^ primary ECs in EGM‐2 medium. As reference material, endothelialized hollow fiber membranes (HFM) prepared as previously described [32] were punched from larger membrane sheets using an 8 mm biopsy punch (pfm medical, Germany) and transferred into 12‐well plates. After 72 h of incubation, cell viability was assessed using live/dead fluorescence staining. Culture medium was replaced with EGM‐2 containing 1.5 µM Calcein‐acetoxymethyl ester (AM) (Thermo Fisher Scientific, Germany) for detection of viable ECs and 2 µM Ethidium Homodimer‐1 (Thermo Fisher Scientific, Germany) to identify non‐viable cells. Following 30 min incubation, staining solution was replaced with fresh medium and samples were analyzed using a fluorescence stereomicroscope (Stereo Discovery V8, Zeiss, Germany). Images were acquired using an AxioCam Icm1 camera (Zeiss, Germany).

#### 2.5.4 Platelet Adhesion Assay

Hemocompatibility was assessed by quantification of platelet adhesion. Platelets were obtained by plateletpheresis from anonymous healthy donors at the blood bank of Hannover Medical School (Hannover, Germany) following informed consent. Platelet concentrates were diluted 1:10 in Tyrode's buffer supplemented with 15 ng mL^−1^ iloprost (Ventavis, Bayer Vital GmbH, Germany) to prevent undesired platelet activation and centrifuged at 800 × g for 15 min without brake. The platelet pellet was resuspended in Tyrode's buffer, pooled from six donors, and stained with 4 µg mL^−1^ 2',7'‐bis‐(2‐Carboxyethyl)‐5‐(and‐6)‐carboxyfluorescein, acetoxymethyl ester (BCECF‐AM, Invitrogen, Germany) for 1 h at 37°C under gentle rotation. After staining, platelets were washed three times to remove excess dye and subsequently resuspended in EGM‐2 medium. For adhesion experiments, punched HFM samples (8 mm diameter), 3D‐printed material specimens, and human plasma‐coated glass coverslips (Haemochrom Diagnostica GmbH, Germany) as positive control were placed in 12‐well plates. Samples were incubated with 1 mL platelet suspension (1 × 10^8^ platelets cm^−^^2^) for 1 h on a rocking platform. Following incubation, samples were gently rinsed with Dulbecco's phosphate‐buffered saline (DPBS) to remove non‐adherent platelets and transferred into lysis buffer (0.1% SDS in 30 mM tris(hydroxymethyl)aminomethane (Tris), pH 8.8). After 30 min of lysis, released fluorescence dye was quantified using a microplate reader (Synergy‐2, BioTek, Germany) at 485 nm excitation and 535 nm emission. Fluorescence values were converted to absolute platelet numbers using a standard calibration curve. Platelet adhesion was normalized to the geometrical projected surface area of the samples without correction for material porosity. Results are presented as mean ± standard deviation from independent triplicates.

#### 2.5.5 Gene Expression Analysis of Endothelial Activation Markers

The endothelial activation state on xPDMS was evaluated by quantitative real‐time polymerase chain reaction (PCR) after 72 h of cultivation on fibronectin‐coated substrates. Total ribonucleic acid (RNA) was isolated from ECs cultured on fibronectin (FN)‐coated xPDMS (see 2.6.1) and from control ECs grown on glass coverslips under standard culture conditions, serving as material‐independent reference. As a positive activation control, ECs were stimulated with 10 ng mL^−1^ tumor necrosis factor alpha (TNFα) (Bachem, Switzerland) for 6 h prior to RNA isolation. RNA extraction was performed using the NucleoSpin II kit (Macherey–Nagel, Germany) according to the manufacturer's instructions. Equal amounts of RNA were reverse‐transcribed using random hexamer primers and the RevertAid H Minus First Strand complementary deoxyribonucleic acid (cDNA) Synthesis Kit (Thermo Fisher Scientific). Quantitative PCR was carried out using SYBR Green Master Mix (Thermo Fisher Scientific) and gene‐specific primers (Table S1) on a LightCycler 96 system (Roche, Germany). Expression levels of endothelial activation markers (E‐selectin, ICAM‐1, VCAM‐1) and coagulation‐associated genes (tissue factor, thrombomodulin) were analyzed using 1:10 diluted cDNA. Relative gene expression was calculated using the ΔCt method (2^−^^ΔCt^), where the cycle threshold (Ct) values were normalized with the respective Ct of the housekeeping gene β‐actin. Primer specificity was confirmed by melting curve analysis, and technical duplicates were performed for each sample.

### 2.6 Biohybrid Lung Integration and Computed Tomography (CT)‐Derived Implant Design

#### 2.6.1 Fibronectin Functionalization of xPDMS

Based on the preceding biocompatibility analyses, FlexaGrey‐PDMS and xPDMS were identified as potential material candidates for TPMS‐based 3D lung constructs. However, simulation results (see Sections 2.2.1 and 3.2) indicated that membrane thicknesses of 10–100 µm are required to achieve oxygen transfer comparable to or exceeding that of conventional HFM oxygenators. The 10 µm membrane thickness represents a theoretical lower boundary derived from gas transport simulations rather than the current additive manufacturing limit. Due to the comparatively thick PDMS coating layer observed for FlexaGrey (>250 µm; Figure 3), this material was not further considered for TPMS fabrication. Although xPDMS exhibited moderately increased platelet adhesion compared to FlexaGrey‐PDMS, previous studies and preliminary data suggest that surface endothelialization can substantially reduce platelet interaction by establishing a physiologically active endothelial barrier [38]. Endothelialization was therefore implemented to assess the potential for functional hemocompatibility enhancement. Analogous to previously described endothelialization strategies for hydrophobic polymer membranes, xPDMS surfaces were functionalized by fibronectin (FN) coating to promote endothelial cell adhesion. Samples were immersed in FN solution (25 µg mL^−1^ in PBS, Corning, Germany) for 2 h at room temperature, transferred to 12‐well plates, and seeded with 5 × 10^4^ ECs in EGM‐2 medium. After 72 h of cultivation, endothelial coverage and viability were assessed using calcein‐AM and ethidium homodimer‐I staining, followed by fluorescence microscopy (Stereo Discovery V8, Zeiss, Germany).

#### 2.6.2 Immunofluorescence Analysis of Junctional and Basement Membrane Proteins

Endothelial phenotype and monolayer integrity on xPDMS were further characterized by immunofluorescence staining of junctional and basement membrane proteins. Endothelialized samples were cultured for 3 days under standard conditions with medium renewal on day 2. Samples were gently rinsed with DPBS and fixed in 4% paraformaldehyde for 5 min at room temperature. Following fixation, cells were permeabilized and blocked using 0.25% Triton X‐100 in Tris‐buffered saline containing 5% donkey serum for 20 min at room temperature. Samples were washed three times with phosphate‐buffered saline (PBS) and incubated with primary antibodies diluted in 1% bovine serum albumin (BSA) in PBS for 1 h at room temperature. Primary antibodies included rabbit anti‐human VE‐cadherin (Abcam, 3.3 µg mL^−^^1^) and mouse anti‐collagen IV (Agilent, 1:50). After washing, fluorescence‐labeled secondary antibodies (donkey anti‐mouse Alexa Fluor 488 and donkey anti‐rabbit Cy3, Jackson ImmunoResearch, UK) were applied for 1 h at room temperature in the dark. Cell nuclei were counterstained with Hoechst 33342 (10 µg mL^−^^1^) during the final 30 min of secondary antibody incubation. Isotype‐matched antibodies served as negative controls. Fluorescence imaging was performed using a confocal laser scanning microscope (CLSM SP8, Leica Microsystems, Germany). Z‐stacks consisting of ≥10 optical sections per region were acquired and processed as maximum intensity projections using ImageJ.

#### 2.6.3 Endothelialization of TPMS‐Based 3D Constructs

Three‐dimensional endothelialization was investigated in TPMS‐based xPDMS constructs to evaluate the feasibility of establishing a continuous endothelial lining throughout the complex internal architecture. Gyroid TPMS constructs comprising 2 × 2 × 2 unit cells (unit cell dimensions 750 µm × 750 µm × 750 µm; X × Y × Z) were fabricated from xPDMS using a Sonic Mini 8K printer (Phrozen, Taiwan). The printed lattice exhibited a structural wall thickness of 1 mm and a printing layer resolution of 100 µm. Following fabrication, constructs were post‐cured for 10 min at 405 nm and sequentially washed in distilled water and ethanol to remove residual monomers. To promote endothelial cell adhesion, xPDMS surfaces were functionalized by FN coating. TPMS constructs were placed in 50 mL syringes (B. Braun, Germany) filled with FN solution (2.5 µg cm^−^^2^ in PBS) and incubated for 2 h at room temperature. Following an established dynamic seeding protocol [28], the coating solution was replaced with EGM‐2 containing 1.5 × 10^5^ ECs cm^−^^2^. Constructs were subjected to rotational incubation (1 rpm) for 24 h at 37°C to promote homogeneous endothelial cell distribution within the three‐dimensional structure, followed by 24 h of static culture. During the static phase, gas exchange was maintained via a sterile filter at the syringe inlet, and culture medium was refreshed four times during the incubation period. For qualitative assessment of endothelial coverage, constructs were fixed in 4% paraformaldehyde and processed for immunofluorescence staining of VE‐cadherin to visualize endothelial intercellular junctions. Cell nuclei were counterstained with Hoechst 33342. Confocal laser scanning microscopy (Leica SP8, Leica Microsystems, Germany) was used to acquire z‐stacks comprising 250 optical sections (10× objective; step size 14.15 µm). Maximum intensity projections were generated using Fiji (ImageJ) for two‐dimensional visualization.

#### 2.6.4 CT‐Based TPMS Implant Design

Computed tomography (CT) data of the rat lung were used to generate individualized, anatomically derived TPMS‐based implant geometries. Two design scenarios were investigated, representing replacement of either the entire right lung or the right upper lobe. The resulting half‐lung implant design exhibited a maximal dimension of approximately 29 mm (tip‐to‐tip), whereas the upper‐lobe implant design measured approximately 16 mm. Depending on the selected TPMS unit‐cell size, the corresponding effective membrane surface areas ranged from 1823–7287 mm^2^ for the half‐lung design and from 501–2005 mm^2^ for the upper‐lobe configuration. The available implant volume was determined as previously described [64].The resulting anatomical volume was defined by a solid housing wall and subsequently filled with membrane structures composed of Schwarz Diamond (SWD) TPMS unit cells with a characteristic size of 1 mm, generated using custom MATLAB code (MathWorks, USA). Two configurations for blood and sweep gas inlet and outlet ports were implemented: a central configuration and a peripheral configuration with apical and basal connections. These configurations were selected to evaluate the influence of port positioning on flow homogeneity within the anatomically derived TPMS implant geometries. Geometric feature design and assembly of all components were performed using Boolean operations in ANSYS SpaceClaim (ANSYS Inc., USA). For flow analysis, the blood domain of each implant design was extracted, and internal flow distribution was simulated using the same CFD settings applied for the velocity analyses described above. Flow rates of 100 and 150 mL min^−1^ were applied, corresponding to the reported cardiac output range of the rat [65]. Gas species transport was not included in these simulations, as the objective of this analysis was to evaluate the flow distribution and perfusion characteristics within the CT‐derived TPMS implant geometries.

### 2.7 Statistics

Statistical analyses were performed using GraphPad Prism (version 9.0, GraphPad Software, USA). For comparisons between two groups, an unpaired two‐tailed Student's *t* test was applied. For multiple group comparisons, one‐way analysis of variance (ANOVA) followed by Dunnett's post hoc test was used. Data are presented as mean ± standard deviation (SD) obtained from independent triplicate experiments (oxygen permeability measurements, platelet adhesion assay, quantitative real‐time PCR) or quadruplicate experiments in the case of extract cytotoxicity testing. Statistical significance was defined as *p* < 0.05 (*), *p* < 0.01 (**), *p* < 0.001 (***), and *p* < 0.0001 (****).

## 3 Results

The following sections investigate how membrane architecture defines flow behavior, gas exchange performance, material requirements for additive manufacturing, and the feasibility of biohybrid triply periodic minimal surfaces (TPMS)‐based lung constructs.

### 3.1 Architecture‐Driven Flow Behavior in TPMS Membrane Structures

Conventional hollow fiber membrane (HFM) oxygenators rely on densely packed fiber bundles (Figure 1a) that create complex interstitial flow paths between individual fibers (Figure 1c). In contrast, TPMS architectures form continuous three‐dimensional membrane structures that generate interconnected flow channels throughout the construct (Figure 1b). Computational fluid dynamics (CFD) simulations were performed to investigate how membrane architecture influences perfusion behavior in artificial lung systems. Velocity fields within HFM bundles revealed pronounced local constrictions between adjacent fibers. These bottlenecks accelerated flow between parallel fibers and generated low‐velocity regions downstream of individual fibers, with stagnation occurring in narrow interstitial regions (Figure 1c). In contrast, TPMS geometries showed a strongly reduced fraction of low‐velocity regions at the applied stagnation threshold and more homogenous flow patterns throughout the computational domain (Figure 1c). Although mean velocities were comparable (0.030 m s^−1^ for HFM and 0.034 m s^−1^ for TPMS), the maximum velocity in HFM reached 0.126 m s^−1^, nearly twice that observed in TPMS (0.062 m s^−1^), indicating substantially broader velocity distributions in HFM architectures. Quantitative analysis of velocity magnitude distributions further confirmed improved flow homogeneity in TPMS structures (Figure 1d). Histogram analysis demonstrated a shift from low‐velocity accumulation in HFM bundles toward more uniformly distributed flow velocities in TPMS architectures. In particular, TPMS structures reduced the fraction of regions below the stagnation threshold (v < 1 × 10^−^^3^ m s^−^^1^) by up to 56% and exhibited an approximately 10% lower standard deviation of the velocity magnitude distribution compared to HFM geometries. Together, these results establish membrane topology as a key design parameter governing perfusion behavior and motivate TPMS‐based constructs as an architecture‐driven platform for biohybrid lung development.

**FIGURE 1:**
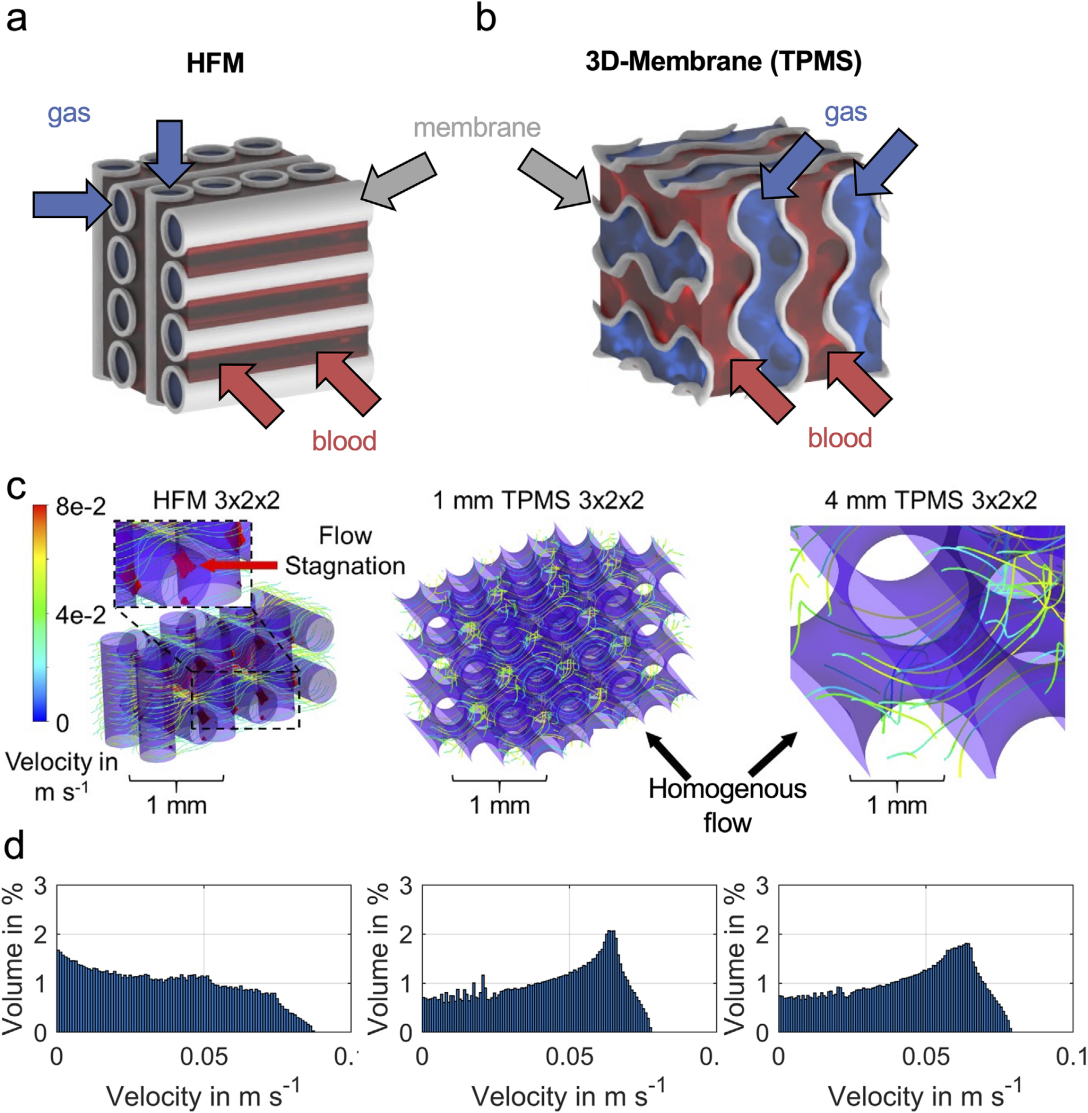
Architecture‐driven flow behavior in TPMS membrane structures. (a) Conceptual comparison of conventional hollow fiber membrane (HFM) bundles, where gas exchange occurs along densely packed parallel fibers forming interstitial flow paths. (b) Triply periodic minimal surface (TPMS) membrane architecture forming a continuous three‐dimensional network with distributed flow channels. (c) Computational fluid dynamics (CFD) simulations showing velocity magnitude fields for HFM bundles and TPMS architectures with unit cell sizes of 1 and 4 mm (3 × 2 × 2 unit cell domain). Regions below the stagnation threshold (v < 1 × 10^−^^3^ m s^−^^1^) are highlighted in red. These simulations illustrate how membrane architecture directly governs perfusion homogeneity within artificial lung systems. (d) Histograms of fluid volume fractions as a function of local flow velocity magnitude for the respective membrane geometries, demonstrating pronounced low‐velocity accumulation in HFM bundles and more homogeneous velocity distributions in TPMS architectures.

### 3.2 Architecture‐Dependent Gas Exchange Performance

Beyond flow homogeneity, membrane architecture also directly influences gas exchange performance. Since local mass transport in artificial lungs strongly depends on perfusion characteristics, the improved flow distribution observed in TPMS architectures is expected to affect oxygen and carbon dioxide transfer. Therefore, architecture‐dependent gas exchange was investigated using multiphysics simulations. Simulation results identified two key design parameters determining the area‐specific gas transfer rate of TPMS membranes: membrane wall thickness and unit cell size. Reducing membrane thickness lowered transmembrane mass transfer resistance for O_2_ and CO_2_, thereby enhancing transmembrane diffusion. Decreasing unit cell size reduced characteristic channel dimensions and distributed the same volumetric blood flow across a larger number of parallel flow paths. Accordingly, decreasing membrane thickness and unit cell size enhanced the flow dependence of area‐specific gas transfer. HFM exhibited mean area‐specific gas transfer rates of 1.41 × 10^−^^5^ m^3^ s^−^^1^ m^−^^2^ for O_2_ and 3.56 × 10^−^^6^ m^3^ s^−^^1^ m^−^^2^ for CO_2_, averaged across all simulated flow rates (Figure 2). Relative values are reported as the 25th percentile, mean, and 75th percentile across all simulated flow rates. TPMS membranes with 10 µm wall thickness and ≤1 mm unit cell size markedly outperformed HFM in oxygen transfer across the investigated flow regime, exhibiting relative increases of +69.3%, +87.8%, and +90.2% for 25th percentile, mean, and 75th percentile values, respectively, compared to HFM. At the same time, CO_2_ transfer remained largely comparable to HFM systems, with relative differences of −5.6%, +3.4%, and +4.5% for the respective percentile values. These findings indicate that architectural optimization affected O_2_ and CO_2_ transfer differently under the investigated conditions. Configurations with a 4 mm unit cell size and 10 µm membrane thickness (−11.3%, −4.8%, −4.0%), as well as a 1 mm unit cell size with 100 µm wall thickness (−32.7%, +8.65%, +16.8%), exhibited oxygen transfer rates comparable to the HFM benchmark. For the optimal TPMS configuration (1 mm unit cell, 10 µm membrane thickness), the required membrane surface area to achieve equivalent oxygen transfer could be reduced by approximately 44.1%, while maintaining comparable CO_2_ transfer (+3.1%) relative to HFM systems. Together, these findings demonstrate that gas exchange efficiency is governed not only by membrane surface area, but also by the three‐dimensional membrane topology controlling local flow distribution and transport behavior (Figure 2).

**FIGURE 2:**
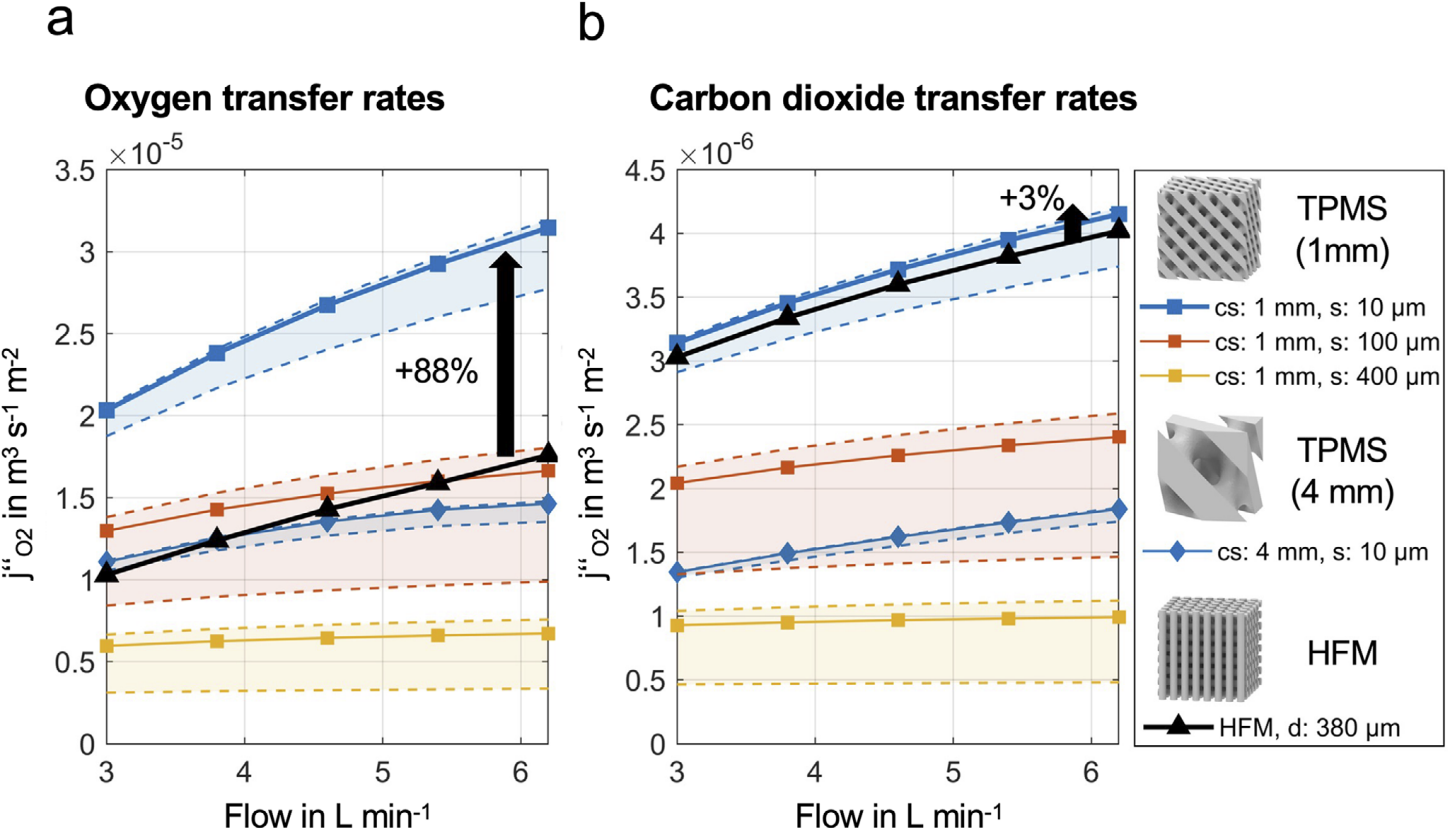
Architecture‐dependent gas exchange performance of TPMS membrane structures. (a) Simulated area‐specific oxygen transfer rates (j″) for hollow fiber membrane (HFM, outer diameter d: 380 µm, membrane wall thickness: 90 µm) and triply periodic minimal surface (TPMS) architectures as a function of flow rate. TPMS structures with unit cell sizes of 1 and 4 mm were evaluated for membrane wall thicknesses of 10, 100, and 400 µm. (b) Corresponding simulations for carbon dioxide transfer under identical conditions. Solid lines represent simulations using the median membrane diffusivity, while dashed lines indicate simulations performed using the 25th and 75th percentile membrane diffusivity bounds used for uncertainty quantification. Symbols distinguish membrane architectures (HFM and TPMS), and colors denote membrane wall thicknesses. While optimized TPMS configurations substantially enhanced O_2_ transfer relative to HFM, CO_2_ transfer remained comparatively stable across architectures, indicating different dominant transport limitations for both gases.

### 3.3 Additive Manufacturing Materials for TPMS Membrane Fabrication

While numerical simulations demonstrated the potential of TPMS architectures to enhance gas exchange performance, translation of these concepts into physical membrane structures requires suitable manufacturing materials and fabrication strategies. Therefore, additive manufacturing‐compatible materials were evaluated with respect to their morphology, structural integrity, and suitability for TPMS membrane fabrication. Selected printable materials for TPMS membrane fabrication were evaluated with respect to surface morphology and coating behavior (Figure 3a–c). Dense polymers methylsiloxane‐based PDMS (MAP‐PDMS) and methacrylate‐functionalized crosslinkable PDMS (xPDMS) exhibited homogeneous, non‐porous surfaces. Notably, xPDMS enabled reproducible fabrication of membrane structures with a uniform thickness of 100 µm throughout the construct, as confirmed by cross‐sectional analysis (Figure S1). Additional scanning electron microscopy (SEM) and energy‐dispersive x‐ray (EDX) analyses further confirmed the dense membrane morphology and homogenous silicon distribution of xPDMS throughout the printed structure. FlexaGrey and PorePro displayed porous architectures requiring subsequent sealing to ensure reliable blood–gas compartment separation. FlexaGrey, a thermoplastic polyurethane (TPU)‐based powder material processed via selective laser sintering (SLS), formed a macroporous network with pore diameters of 10–100 µm and a porosity of 28%. In contrast, PorePro was fabricated via photo‐induced phase separation, resulting in a microporous structure with a mean pore diameter of approximately 170 nm and a porosity of 45%. Establishment of a plasma‐tight barrier while preserving structural integrity required polydimethylsiloxane (PDMS) coating of the porous substrates. EDX mapping confirmed that coating morphology strongly depended on surface pre‐treatment. Oxygen plasma activation followed by ethanol or isopropanol wetting resulted in partial PDMS infiltration into the macroporous FlexaGrey structure (Figure 3c). In contrast, pre‐wetting with deionized water produced a continuous PDMS surface layer with minimal pore penetration (Figure 3c). Cross‐sectional imaging revealed a mean PDMS coating thickness of 403.7 ± 44.9 µm for FlexaGrey, reflecting deeper infiltration into its macroporous structure, whereas PorePro exhibited a thinner and more uniform PDMS layer of 168.2 ± 6.8 µm (Figure 3a). These results highlight that successful implementation of TPMS‐based gas exchange structures requires materials that combine high gas permeability with manufacturability and structural integrity. Material selection therefore represents a key enabling factor for translating architected membrane concepts into physically realizable constructs. Compared to coated PorePro and FlexaGrey structures, xPDMS exhibited substantially improved thickness controllability and morphological precision. This enhanced structural control further supports its suitability as a preferred membrane material for TPMS‐based gas exchange architectures.

**FIGURE 3:**
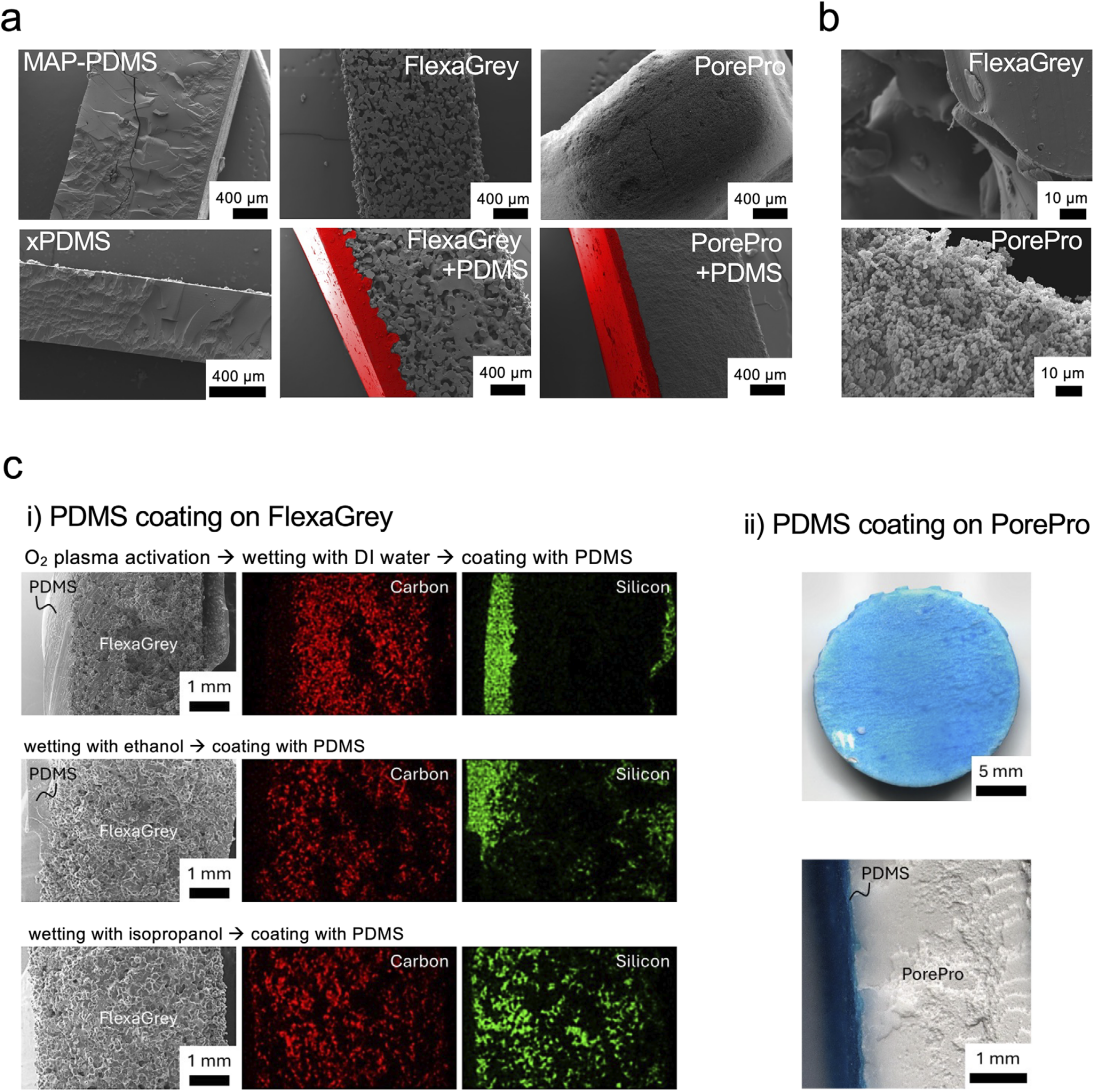
Scanning electron microscopy (SEM) images of cross‐sectioned samples with and without PDMS coating. (a) MAP‐PDMS and xPDMS exhibit dense, non‐porous morphologies, whereas FlexaGrey and PorePro display porous structures requiring subsequent PDMS coating (highlighted in red) to establish a plasma‐tight barrier. Further microscopical analysis of xPDMS with focus on layer thickness and EDX can be found in the Figure S1. (b) FlexaGrey shows macroporous structures with larger pore sizes compared to the microporous morphology of PorePro. (c) PDMS coating. (i) SEM images of PDMS coated FlexaGrey samples. From left to right: SEM image, EDX image showing carbon content, EDX image showing silicon content. Carbon mainly corresponds to the FlexaGrey polymer and silicon visualizes the PDMS. (ii) Microscope images of PDMS coated PorePro, where the PDMS was colored in blue with food dye. The top picture shows the sample, where the entire surface is coated. The bottom picture shows the cross section.

### 3.4 Oxygen Permeability of PDMS‐Based Membrane Materials

Beyond structural manufacturability, membrane materials must provide sufficient gas permeability to enable efficient transmembrane transport. Therefore, oxygen permeability of the investigated materials was quantified and compared to clinical HFM references. Oxygen permeability measurements were performed on disc‐shaped material samples (diameter: 20 mm, 2 mm thickness) to enable standardized comparative assessment. Oxygen permeability measurements revealed immediate gas equilibration for uncoated FlexaGrey and PorePro samples, consistent with their open porous morphology. These materials were therefore excluded from quantitative permeability comparison. All remaining materials were compared to the clinical HFM material (Figure 4), which exhibited an oxygen permeability of 4.06 × 10^−^^1^ ^5^ ± 8.08 × 10^−^^1^ ^7^ mol m m^−^^2^ s^−^^1^ Pa^−^^1^, consistent with literature values [66]. In comparison, PDMS demonstrated significantly higher oxygen permeability (2.17 × 10^−^^1^ ^3^ ± 2.32 × 10^−^^14^ mol m m^−^^2^ s^−^^1^ Pa^−^^1^, *p* = 0.0079). Similarly elevated values were observed for MAP‐PDMS (2.13 × 10^−^^1^ ^3^ ± 1.99 × 10^−^^14^ mol m m^−^^2^ s^−^^1^ Pa^−^^1^, *p* = 0.0091) and PDMS‐coated PorePro (3.43 × 10^−^^1^ ^3^ ± 1.53 × 10^−^^1^ ^3^ mol m m^−^^2^ s^−^^1^ Pa^−^^1^, *p* = 0.0294). Although not reaching statistical significance, xPDMS and PDMS‐coated FlexaGrey also demonstrated substantially higher oxygen permeability compared to HFM. These results confirm that PDMS‐based materials provide substantially higher intrinsic oxygen permeability than conventional HFM, supporting their suitability for thin membrane architectures required for TPMS‐based gas exchange structures.

**FIGURE 4:**
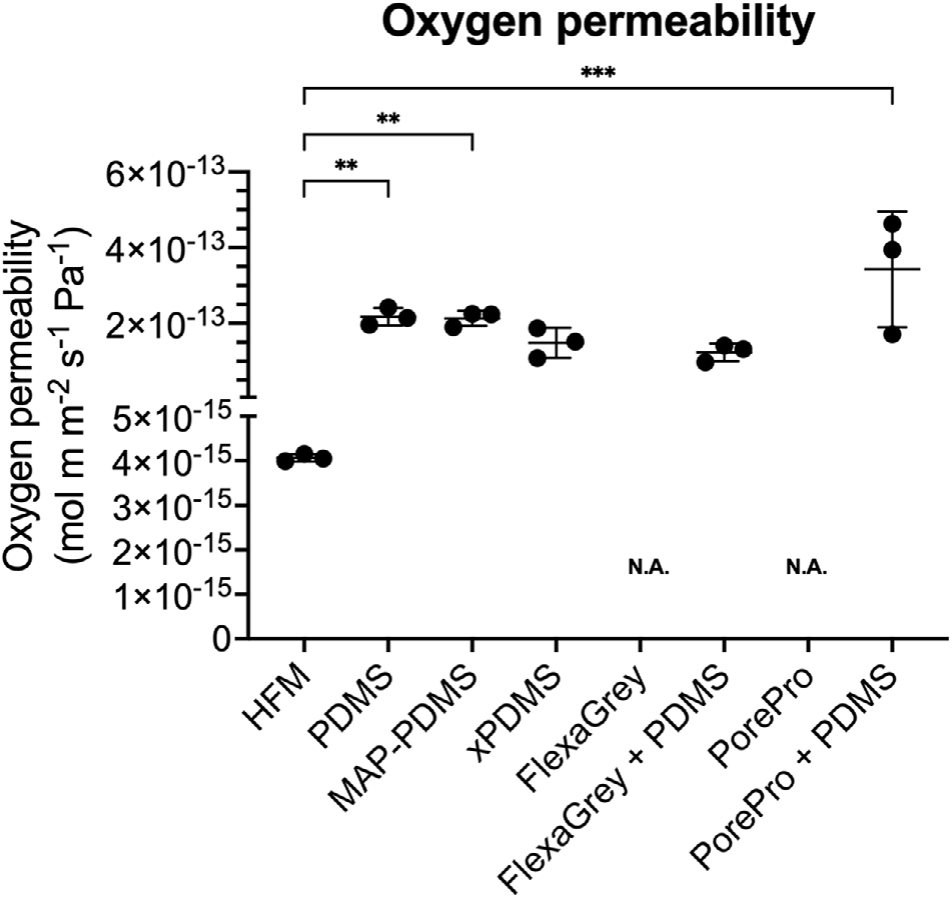
Oxygen permeability of PDMS‐based materials and PDMS‐coated porous substrates compared to clinical hollow fiber membranes. Measurements were performed using disc‐shaped samples (20 mm diameter, 2 mm thickness). Uncoated FlexaGrey and PorePro samples were excluded due to immediate gas equilibration through their open porous structures (N.A., not applicable). Data represent mean permeability values from three independent samples reported in SI units. Statistical differences relative to PMP were evaluated using one‐way ANOVA followed by Dunnett's multiple comparison test (*p < 0.05, **p < 0.01, ***p < 0.001).

### 3.5 Biocompatibility and Hemocompatibility of TPMS Membrane Materials

In addition to gas transport properties, materials intended for artificial lung applications must exhibit high biocompatibility and hemocompatibility when exposed to blood components. Therefore, endothelial cell compatibility and platelet adhesion were evaluated for the investigated membrane materials. Extract‐based cytotoxicity testing revealed material‐dependent differences in EC viability (Figure 5a). MAP‐PDMS extract induced pronounced cytotoxicity, reducing EC viability to 8.96 ± 5.58% (*p* < 0.0001). PorePro extract also significantly decreased viability to 52.37 ± 22.38% (*p* = 0.0002). Following PDMS coating, PorePro exhibited increased viability (85.70 ± 12.14%), approaching the ISO cytotoxicity threshold. In contrast, extracts of HFM, xPDMS, FlexaGrey, PDMS‐coated FlexaGrey, and pure PDMS maintained EC viability above the ISO‐defined cytotoxicity limit, although the standard deviation for pure PDMS approached the cytotoxicity threshold. Direct EC seeding confirmed these findings (Figure 5b). xPDMS and PDMS‐coated FlexaGrey supported dense coverage of viable ECs, whereas PDMS, MAP‐PDMS, and PDMS‐coated PorePro exhibited sparse viable cells accompanied by non‐viable nuclei. Platelet adhesion to the material surfaces was quantified to assess hemocompatibility (Figure 5c). HFM served as reference (2.35 × 10^6^ ± 0.29 × 10^6^ platelets cm^−^^2^), while a positive control represented maximal platelet adhesion (1.33 × 10^8^ ± 8.66 × 10^6^ platelets cm^−^^2^). PDMS‐based materials exhibited markedly reduced platelet adhesion compared to the positive control. Adhesion levels for PDMS and MAP‐PDMS were comparable to HFM, whereas xPDMS showed moderately elevated platelet adhesion relative to the reference (2.95 × 10^7^ ± 0.91 × 10^7^ platelets cm^−^^2^). PDMS coating of porous substrates significantly reduced platelet adhesion compared to the corresponding uncoated materials. In particular, FlexaGrey‐PDMS exhibited adhesion levels comparable to HFM and pure PDMS. Considering its favorable combination of printability, gas transport properties, and cytocompatibility/hemocompatibility, xPDMS was selected as the candidate membrane material for TPMS‐based constructs and subjected to further functional evaluation of endothelial phenotype. Gene expression analysis demonstrated that ECs cultured on fibronectin (FN)‐coated xPDMS maintained a non‐activated endothelial phenotype, with expression of E‐selectin, ICAM‐1, and VCAM‐1 comparable to non‐activated control cells and significantly lower than tumor necrosis factor alpha (TNFα)‐stimulated ECs (*p* < 0.0001) (Figure 5d). Expression of the pro‐thrombogenic marker tissue factor remained unchanged relative to controls, whereas the anti‐thrombogenic marker thrombomodulin was significantly elevated in ECs cultured on FN‐coated xPDMS. These findings demonstrate that xPDMS provides a suitable biological interface for endothelialized TPMS membranes and establishes the basis for subsequent biohybrid integration of architecture‐driven TPMS gas exchange structures.

**FIGURE 5:**
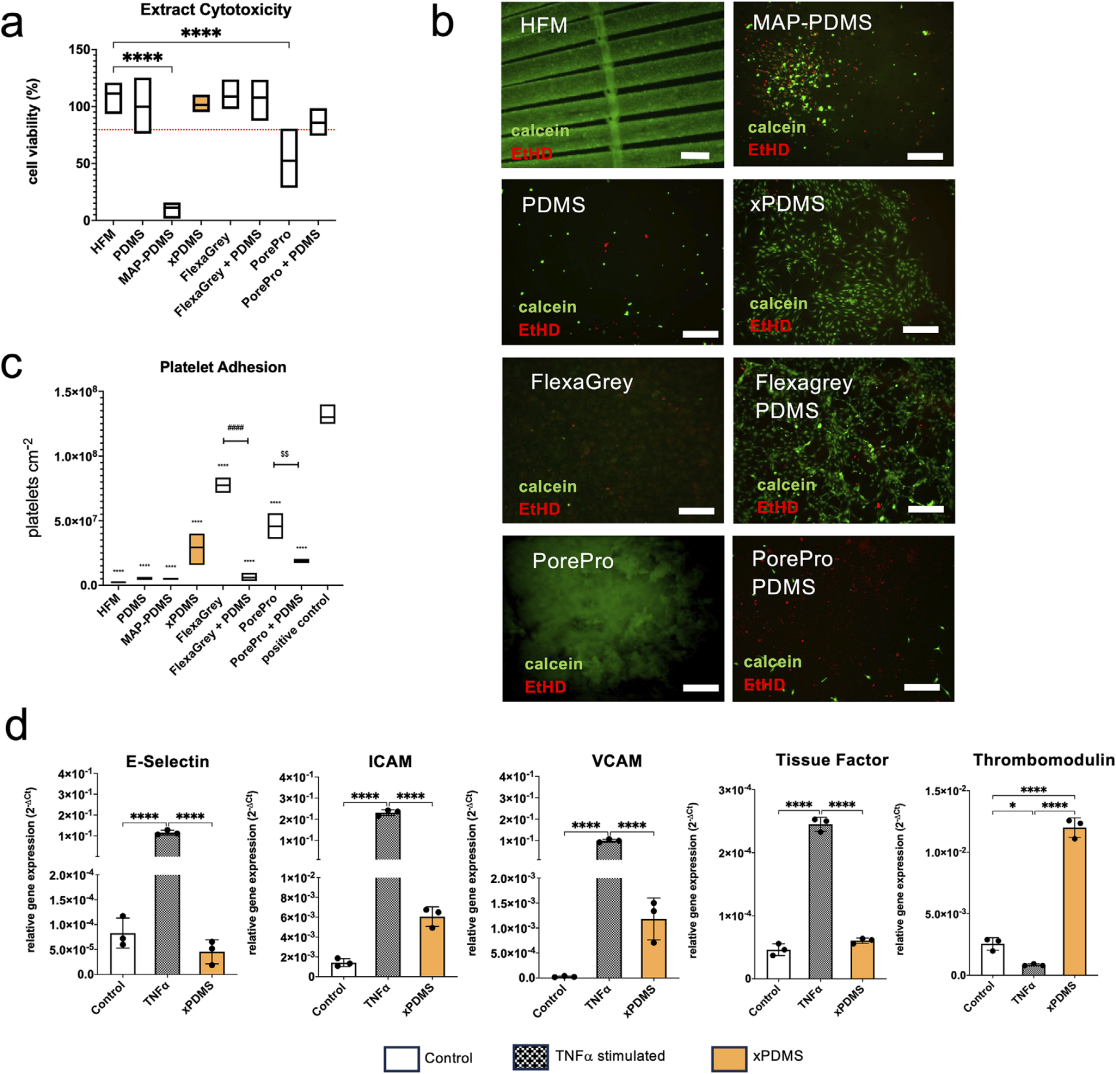
Biocompatibility and hemocompatibility of selected membranes. (a) Cytotoxicity of extracts derived from three‐dimensional (3D)‐printed materials with and without PDMS coating. Endothelial cells (ECs) were incubated with material extracts for 24 h and metabolic activity was quantified using the WST‐8 assay. Viability is expressed relative to ECs cultured in standard medium (100%). The red dotted line indicates the ISO 10993‐5 cytotoxicity threshold (80% viability). Data represent mean ± SD (*n* = 4). Statistical differences relative to heparin/albumin‐coated hollow fiber membranes (HFM) were evaluated using one‐way ANOVA followed by Bonferroni's multiple comparison test. (b) Endothelial cell adhesion on material surfaces. Fluorescence microscopy images of live/dead staining after 72 h of cultivation. Viable ECs are stained with calcein (green), whereas nuclei of non‐viable cells are stained with ethidium homodimer‐1 (EtHD‐1, red). Scale bar: 400 µm. (c) Platelet adhesion on selected membrane materials. Platelet adhesion experiments were performed on disc‐shaped material samples and punched HFM specimens (8 mm biopsy punch diameter). Fluorescence‐labeled platelets adhering to the material surfaces after 1 h incubation were quantified per surface area. Data represent mean ± SD from four independent experiments (*n* = 4). Statistical differences relative to the positive control were assessed using one‐way ANOVA followed by Dunnett's multiple comparison test. Direct comparisons between coated and uncoated porous substrates were analyzed using Student's *t*‐test (####*p* < 0.0001 for FlexaGrey vs. FlexaGrey‐PDMS; $$ *p* < 0.01 for PorePro vs. PorePro‐ PDMS). (d) Gene expression analysis of endothelial activation markers determined by qRT‐PCR. Expression levels of endothelial activation markers were analyzed in ECs cultured on fibronectin‐coated xPDMS and analyzed relative to control ECs cultured on standard tissue culture plastic, with or without TNFα stimulation (6 h). Data were analyzed using one‐way ANOVA followed by Tukey's multiple comparison test.

### 3.6 Biohybrid Integration and CT‐Derived Lung Implant Design

Building on the favorable biological interface established for xPDMS‐based TPMS membranes, endothelialization strategies were further investigated to enable biohybrid lung integration. To further enhance hemocompatibility and establish a physiologically functional blood‐contacting surface, endothelialization strategies were investigated as part of a biohybrid lung concept. Fibronectin functionalization improved endothelial coverage on xPDMS, resulting in the formation of a confluent endothelial monolayer. Immunofluorescence analysis confirmed formation of a continuous vascular endothelial (VE)‐cadherin‐positive endothelial monolayer with deposition of collagen IV, indicating development of a basement membrane–associated extracellular matrix (Figure 6a). Endothelialization of TPMS‐based three‐dimensional (3D) constructs demonstrated the feasibility of establishing a continuous endothelial layer along the complex scaffold geometry. Confocal z‐stack imaging revealed interconnected VE‐cadherin‐positive junctions following the curved TPMS architecture (Figure 6b). Finally, computed tomography (CT)‐derived implant designs demonstrated the feasibility of translating anatomical lung volumes into TPMS‐filled implant concepts and enabled evaluation of flow distribution within anatomically derived geometries. Two implantation scenarios (whole right lung vs. right upper lobe replacement) and two port configurations (central vs. apical/basal) were implemented to assess perfusion homogeneity within the anatomically derived constructs (Figure 6c). Together, these results provide proof‐of‐concept for a biohybrid TPMS‐based lung platform integrating membrane architecture, manufacturable materials, endothelial functionalization, and CT‐derived translational implant design.

**FIGURE 6:**
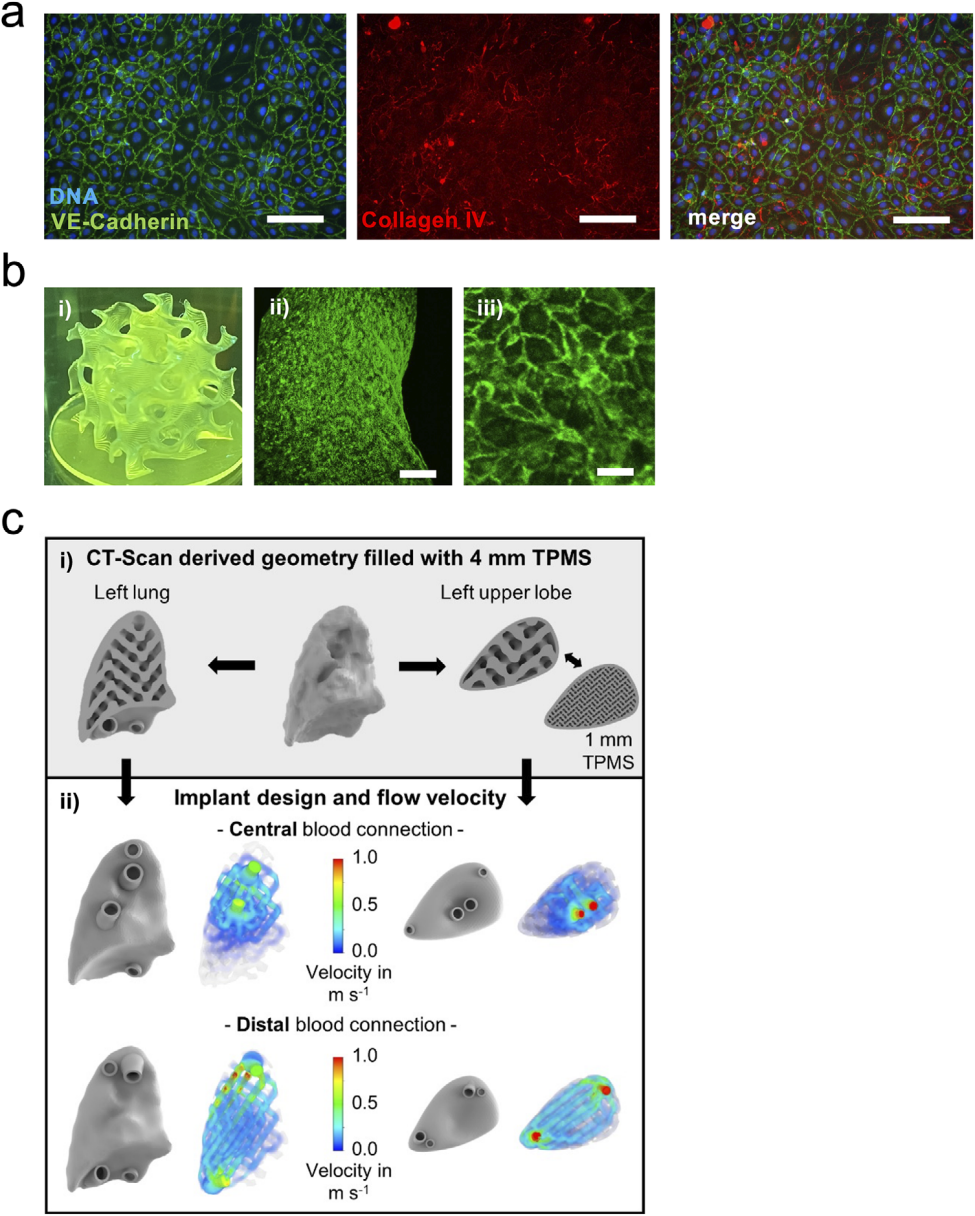
(a) Immunofluorescence analysis of endothelial phenotype on fibronectin‐coated xPDMS. Endothelial cells were stained for VE‐cadherin (green) to visualize intercellular junctions and collagen IV (red) to detect subendothelial extracellular matrix deposition. Nuclei were counterstained with Hoechst 33342 (blue). Continuous VE‐cadherin–positive junctions and collagen IV deposition indicate formation of a confluent endothelial monolayer. Scale bar: 100 µm. (b) Endothelialization of TPMS‐based 3D constructs. Confocal laser scanning microscopy images of endothelial cells cultured on fibronectin‐coated xPDMS TPMS scaffolds. (i) Top‐view projection showing homogeneous endothelial coverage of the scaffold surface. (ii) Three‐dimensional reconstruction generated from confocal z‐stacks illustrating endothelial lining along the curved TPMS geometry. Scale bar: 300 µm. (iii) Higher magnification revealing VE‐cadherin–positive intercellular junctions. Scale bar: 30 µm. (c) Translation of computed tomography (CT) data into TPMS‐based lung implant design. (i) CT‐derived reconstruction of the implantable space within the right lung, illustrating two replacement scenarios (entire right lung and right upper lobe). (ii) Design configurations of the TPMS‐based lung implant with blood and sweep gas inlet/outlet ports positioned either centrally or apically/basally. Corresponding CFD simulations showing velocity distributions within the TPMS architecture for each configuration.

## 4 Discussion

### 4.1 Paradigm Shift: From Fiber Bundles to Architected Membranes

Extracorporeal membrane oxygenation systems currently rely almost exclusively on hollow fiber membrane (HFM) bundles, where gas exchange performance is largely constrained by heterogeneous flow distribution and limited control over three‐dimensional perfusion pathways. In this study, we introduce an architecture‐driven design paradigm for artificial lung development in which gas exchange performance is governed primarily by three‐dimensional membrane architecture rather than by incremental optimization of conventional hollow fiber bundle systems. Using triply periodic minimal surfaces (TPMS) architectures, we demonstrate how computational design, additive manufacturing‐compatible materials, and endothelial functionalization can be integrated to define a new design space for biohybrid lung constructs. In contrast to discrete fiber bundles, TPMS geometries establish continuous three‐dimensional membrane structures that enable distributed perfusion pathways and more homogeneous flow fields, thereby directly influencing transport phenomena and hemodynamic conditions within the gas exchange unit. These findings establish membrane topology itself as a central regulator of oxygenator performance by simultaneously influencing gas transport, flow homogenization, and thrombogenic risk within a unified structural framework. Notably, TPMS architectures influenced O_2_ and CO_2_ transport differently. While optimized TPMS membranes substantially increased O_2_ transfer, CO_2_ transfer remained largely comparable to conventional HFM systems. This difference can be explained by the distinct relative contributions of convective, diffusive, and blood‐side transport resistances for both gases. TPMS architectures reduce local flow heterogeneity and decrease characteristic diffusion distances, thereby reducing blood‐side boundary‐layer resistance. Quantitative velocity‐distribution analysis further demonstrated that TPMS architectures reduce low‐velocity accumulation and stagnation‐prone regions compared to conventional HFM bundles, consistent with a more homogeneous perfusion behavior. These improvements in flow homogeneity particularly benefit O_2_ transport, which is more sensitive to local diffusion limitation and perfusion heterogeneity. In contrast, CO_2_ transport is less sensitive to architectural flow heterogeneity due to its higher effective solubility and diffusivity, as well as its favorable dissociation behavior in blood. The diffusivity values assigned to PDMS in the present simulations were 1.6 × 10^−^^3^ mm^2^ s^−^^1^ for O_2_ and 2.6 × 10^−^^3^ mm^2^ s^−^^1^ for CO_2_ in PDMS, corresponding to an approximately 60% higher diffusivity for CO_2_ and, consequently, a lower membrane diffusion resistance. Consequently, membrane diffusion resistance contributes proportionally less to the overall mass transfer resistance for CO_2_ than for O_2_. Furthermore, under the investigated conditions, oxygen transport is more strongly limited by diffusion within the blood phase, where the effective diffusivity of O_2_ is substantially lower than that of CO_2_ (2.2 × 10^−^^4^ mm^2^ s^−^^1^ versus 7.4 × 10^−^^4^ mm^2^ s^−^^1^). Therefore, reductions in blood‐side transport resistance achieved by the more homogeneous TPMS flow patterns predominantly enhance O_2_ transport, whereas CO_2_ transfer remains comparatively insensitive because the overall mass transfer resistance for CO_2_ is less dominated by blood‐side diffusion limitations. This mechanistic interpretation is consistent with the simulation results, in which the optimized TPMS configuration increased mean O_2_ transfer by 87.8%, whereas mean CO_2_ transfer increased by only 3.4%. Importantly, the optimal TPMS configuration achieved equivalent oxygen transfer while requiring substantially less membrane surface area compared to conventional hollow fiber systems. Reducing the total blood–material interface area may therefore represent an additional strategy to mitigate thrombogenic interactions in future oxygenator designs. However, implementation of dense TPMS membranes introduces distinct material requirements compared to porous hollow fiber systems. Accordingly, TPMS membranes require thin, gas‐permeable, liquid‐tight materials compatible with additive manufacturing. Among the investigated candidates, methacrylate‐functionalized crosslinkable PDMS (xPDMS) provided the most favorable combination of digital light processing (DLP) printability, oxygen permeability, cytocompatibility, and the ability to support endothelialization. This material compatibility enables integration of TPMS architectures into biohybrid lung concepts combining flow optimization with endothelial functionalization to improve hemocompatibility.

### 4.2 Design Parameters Anchored in Manufacturing Reality

While the previous section introduced the architecture‐driven design concept, practical implementation of TPMS‐based gas exchange structures requires design parameters that remain compatible with current additive manufacturing capabilities. The membrane thicknesses (10, 100, and 400 µm) and TPMS unit cell dimensions (1 and 4 mm) investigated in this study were therefore intentionally bounded by fabrication feasibility rather than theoretical optima. A membrane thickness of 10 µm reflects experimentally achievable limits for dense polydimethylsiloxane (PDMS)‐based membrane layers and approaches the lower boundary for mechanically stable, liquid‐tight elastomeric structures produced via additive manufacturing. For comparison, clinically used HFM consists of a microporous core structure covered by a thin dense outer skin (typically on the order of micrometers), preventing plasma leakage while introducing an additional diffusion barrier inherently coupled to the fiber‐based architecture [67, 68]. In dense TPMS membranes, however, mass transfer resistance must be integrated directly into the structural wall thickness, which requires micrometer‐scale fabrication precision. Similarly, TPMS unit cell dimensions of approximately 1 mm represent the lower limit of reliably printable continuous minimal surface geometries using currently available additive manufacturing platforms, as previously demonstrated for structurally stable TPMS constructs [69]. The 4 mm configuration investigated here reflects a more fabrication‐robust geometry that facilitates homogeneous coating behavior and reproducible manufacturing. By constraining the investigated parameter space to fabrication‐realistic geometries, this study defines a practical design window linking membrane architecture with manufacturability. Within this window, the simulations demonstrate that clinically relevant gas transfer performance can be achieved even when membrane mass transfer resistance is explicitly incorporated into the structural membrane thickness. The experimentally determined oxygen permeability measurements on 2 mm disc‐shaped samples were primarily intended as a comparative material‐screening approach rather than exact representations of final TPMS membrane performance under physiological conditions. Within the solution‐diffusion framework, permeability represents an intrinsic material property, whereas the resulting transmembrane flux scales inversely with membrane thickness. Accordingly, these experiments confirmed the generally high gas permeability of the investigated PDMS‐based materials. Because the measurements were performed at room temperature rather than under physiological conditions, they should be regarded primarily as comparative screening data and may provide a conservative estimate of gas transport under physiological conditions. To account for uncertainty in reported PDMS transport properties, the multiphysics simulations incorporated literature‐based diffusivity ranges including 25th, 50th, and 75th percentile values rather than relying on a single experimentally determined parameter set. Accordingly, the simulations should primarily be interpreted as comparative analyses of architecture‐dependent transport behavior rather than exact predictions of absolute in vivo gas exchange rates. Together, these findings support a design strategy in which membrane architecture, material permeability, and manufacturing resolution are optimized in a coordinated manner rather than through membrane surface area scaling alone.

### 4.3 Material Considerations and Printability Constraints

While the previous section defined the geometry‐manufacturing design window, practical implementation of dense TPMS membranes additionally requires materials compatible with both gas transport and additive manufacturing. In contrast to HFM, where gas transport is enabled by microporous substrates with ultrathin selective outer skins [70], dense TPMS membranes must provide intrinsic gas permeability within the structural membrane itself while maintaining strict separation between blood and sweep gas compartments. PDMS served as a reference material in the simulations due to its high gas permeability and suitability for thin elastomeric membrane fabrication [23, 47]. Since the primary objective of the material screening was comparative evaluation of membrane suitability for oxygenation performance in artificial lung systems, experimental permeability characterization focused on O_2_ transport. Given the higher diffusivity and solubility of CO_2_ in PDMS‐based polymers, major qualitative differences between the investigated materials were not expected. However, conventional PDMS formulations are not directly compatible with UV‐light‐based additive manufacturing. To address this limitation, modified precursor resins (methylsiloxane‐based PDMS (MAP‐PDMS), methacrylate‐functionalized crosslinkable PDMS (xPDMS)) and porous scaffold materials (FlexaGrey, PorePro) subsequently sealed by PDMS coating were evaluated. The additive manufacturing route strongly influenced the achievable membrane architecture. Selective laser sintering (FlexaGrey) enables fabrication of flexible TPU scaffolds but introduces practical limitations for enclosed TPMS geometries, as removal of unsintered powder from complex internal channels becomes challenging. In addition, the large pore sizes of the printed thermoplastic polyurethane (TPU) network promote capillary infiltration of liquid PDMS, resulting in thick and spatially heterogeneous coating layers. In contrast, stereolithography‐based processes (PorePro, xPDMS) provide higher geometric resolution and facilitate removal of unpolymerized resin from enclosed structures. However, photopolymer systems may release cytotoxic leachables associated with residual photoinitiators or unreacted monomers after polymerization [71]. The pronounced cytotoxicity observed for MAP‐PDMS is therefore plausibly attributable to incomplete polymerization or residual dye components used to control UV penetration depth. Interestingly, the improved cytocompatibility observed after PDMS coating of PorePro suggests that the dense elastomer layer may act as an effective barrier against diffusion of low‐molecular‐weight toxic components from the underlying polymer network. Among the investigated candidates, xPDMS provided the most favorable combination of DLP printability, oxygen permeability, cytocompatibility, and endothelialization compatibility within the simulated thickness window. In contrast to secondary PDMS coating approaches on porous scaffold materials, xPDMS enabled direct fabrication of dense gas‐permeable membrane structures with reproducible and spatially well‐defined membrane thicknesses determined by the DLP printing process itself. This improved geometric controllability enables more precise integration of membrane thickness, gas transport, and flow architecture in TPMS oxygenators. Collectively, these findings demonstrate that successful TPMS fabrication requires coordinated optimization of manufacturing resolution, polymer chemistry, and coating strategies. Despite the successful fabrication of simplified TPMS membrane structures, translation toward fully enclosed anatomically derived TPMS lung implants remains associated with substantial manufacturing challenges. In particular, current DLP‐based printing systems remain limited in their ability to simultaneously achieve micrometer‐scale resolution and centimeter‐scale build volumes required for large‐scale TPMS constructs. In addition, post‐processing of xPDMS TPMS lung constructs revealed crack formation during solvent evaporation after washing, likely caused by differential shrinkage between the outer shell and the internal TPMS network during drying. Faster solvent evaporation at the outer shell likely induces heterogeneous shrinkage and internal mechanical stress. Potential solutions may include humidity‐controlled drying strategies to reduce differential shrinkage during solvent evaporation. Modular fabrication approaches with separately manufactured housing and TPMS membrane components may further improve structural stability during post‐processing. In addition, optimization of resin formulations and light‐absorbing dyes may improve printing resolution and enable smaller TPMS unit‐cell dimensions. Indirect additive manufacturing strategies may provide an additional route toward improved structural resolution, as recently demonstrated for 1 mm TPMS membrane architectures.

### 4.4 Architectural Context: Beyond Fibers and Planar Microfluidics

Microfluidic oxygenators fabricated from layered PDMS membranes have demonstrated high gas transfer efficiencies at small scales due to ultrathin membranes (30–120 µm) and short diffusion paths [26]. However, these systems are typically based on planar microchannel architectures and are scaled by stacking or parallelization of discrete layers. Such hierarchical branching networks may increase hydraulic resistance and limit volumetric scalability at clinically relevant blood flow rates, while simultaneously introducing non‐functional blood‐contacting surface area, thereby increasing the overall blood‐contacting interface without proportional functional benefit [23, 27]. In contrast, TPMS architectures establish a continuous three‐dimensional membrane topology in which gas exchange surfaces are distributed throughout a single interconnected flow domain. Unlike planar microchannel systems, TPMS structures enable volumetric scaling within a unified geometry while maintaining continuous perfusion pathways analogous to hollow fiber oxygenators. Thus, while microfluidic devices demonstrate the transport potential of thin PDMS membranes, architected TPMS membranes extend these principles into volumetrically integrated gas exchange structures that are compatible with anatomical adaptation and CT‐based implant design. TPMS architectures therefore bridge the gap between high‐efficiency microfluidic membrane transport and clinically relevant volumetric oxygenator design.

### 4.5 Hemocompatibility and Biohybrid Integration

Beyond architectural optimization of TPMS membranes for volumetric gas exchange, long‐term clinical translation of artificial lung systems ultimately depends on restoring physiological hemocompatibility. Surface endothelialization therefore represents a complementary strategy to geometry‐driven flow homogenization. Endothelialization of blood‐contacting oxygenator surfaces has previously been demonstrated as a viable strategy to improve hemocompatibility [29, 31, 32, 36, 37, 38, 39, 40]. Consistent with these findings, fibronectin‐assisted endothelialization of xPDMS TPMS constructs resulted in viable, confluent endothelial monolayers exhibiting preserved junctional integrity and thrombomodulin expression. While xPDMS alone exhibited moderately elevated platelet adhesion compared to PDMS‐coated FlexaGrey—the most biocompatible porous reference material among the tested substrates—endothelialization effectively re‐established a non‐inflammatory and anti‐thrombogenic phenotype, as indicated by preserved quiescent activation marker expression and enhanced thrombomodulin levels. PDMS surface modification via oxygen plasma treatment is known to transiently increase surface hydrophilicity but typically lacks long‐term stability [72, 73]. Extracellular matrix protein coating was therefore employed to promote stable endothelial adhesion and maintain monolayer integrity on the TPMS structures. Additional surface‐engineering strategies such as covalent heparinization, polyethylene glycol attachment ((PEG)ylation), or zwitterionic surface modifications may further improve hemocompatibility of TPMS‐based artificial lung systems, although such approaches must be balanced against potential effects on gas permeability and endothelial adhesion. Three‐dimensional endothelial coverage of the architected TPMS geometries further demonstrates that minimal surface membranes are not only printable but also allow biological functionalization. While the present study demonstrates successful establishment of confluent endothelial monolayers on xPDMS‐based TPMS structures, including preserved intercellular junction formation, basement membrane deposition, and low inflammatory activation within the investigated cultivation period, long‐term endothelial stability under chronic flow exposure remains to be established. The present study was designed as a translational proof‐of‐concept focusing on endothelial compatibility and biological integration within architected TPMS geometries during early cultivation phases rather than chronic extracorporeal support conditions. Importantly, the observed expression of vascular endothelial (VE)‐cadherin, collagen IV, and thrombomodulin, together with the low expression of inflammatory activation markers, indicates maintenance of a functionally quiescent endothelial phenotype during the investigated culture period. Future studies will therefore need to investigate long‐term endothelial durability, barrier stability, shear‐dependent remodeling, and phenotypic maintenance under continuous perfusion conditions relevant to chronic biohybrid lung support. This establishes an important translational in vitro validation step toward the biohybrid lung concept in which geometry‐driven flow homogenization and cell‐mediated anticoagulation act synergistically rather than independently. Importantly, the reduced stagnation potential of TPMS architectures [42], combined with endothelial‐mediated anticoagulant regulation, may provide complementary mechanisms to mitigate thrombogenic risk. Even in scenarios of partial endothelial disruption, the more homogeneous flow environment of TPMS membranes may offer intrinsic protection compared to conventional hollow fiber bundles.

### 4.6 Translational Perspective and Future Optimization

Building on the architectural and biological validation of TPMS membranes, clinical translation additionally requires scalable architectures and anatomically compatible implant geometries. The simulations were performed within flow regimes relevant for small‐animal extracorporeal membrane oxygenation (ECMO) models (≤140 mL min^−^^1^), providing a translational entry point for cost‐efficient in vivo validation. Although these flow regimes are below those required for clinical extracorporeal support, the architecture‐driven design principles demonstrated here are inherently scalable and can be extended to higher flow rates through parallelization and geometric scaling of TPMS modules. Translation toward rapid‐prototype ECMO devices and endothelialized biohybrid lung systems will additionally require scalable and reproducible manufacturing and cell‐seeding strategies. Manufacturing challenges include reliable production of thin, defect‐free, liquid‐tight TPMS membranes, removal of uncured resin from enclosed internal channels, quality control of membrane thickness and gas‐tightness across large surface areas, and reproducible sterilization without compromising material properties or endothelial compatibility. For endothelialized TPMS lungs, an additional challenge is the homogeneous distribution and retention of endothelial cells throughout complex three‐dimensional internal geometries. Static seeding is unlikely to be sufficient at larger scales; therefore, dynamic perfusion‐ or rotation‐assisted seeding strategies, as previously established for biohybrid lung endothelialization [28] will likely be required to achieve uniform endothelial coverage throughout complex TPMS geometries. These manufacturing and biological challenges represent critical next steps toward the clinical translation of TPMS‐based lung systems. Progressive scaling toward clinically relevant blood flow rates will require further reductions in membrane thickness and unit cell dimensions, potentially achievable through emerging high‐resolution additive manufacturing techniques such as two‐photon polymerization [74]. While oxygen transfer improvements were pronounced, CO_2_ transfer did not exhibit comparable enhancement. This difference likely reflects the inherently higher diffusivity of CO_2_ and the resulting, smaller concentration gradients where the impact of passive mixing is less prominent. Consequently, oxygen transfer represents the primary performance limitation of current oxygenators, whereas CO_2_ elimination is generally not rate‐limiting. In this context, maintaining CO_2_ transfer comparable to conventional HFM systems may be sufficient when accompanied by reduced stagnation potential and improved flow homogenization. Residual low‐velocity regions observed near housing intersections in the computed tomography (CT)‐derived implant geometries could be further minimized through automated geometry optimization. Although direct multiphysics gas transfer simulations were not performed for the full CT‐derived implant geometries due to the substantial computational costs associated with resolving species transport across anatomically scaled TPMS domains, the computational fluid dynamics (CFD)‐derived flow distributions could be related to the architecture‐dependent transfer characteristics identified in Section 3.2. The resulting analyses demonstrated that peripheral apical‐basal connector positioning improved flow homogenization and increased predicted oxygen transfer rates compared to central connector configurations. In addition, smaller TPMS unit‐cell geometries further enhanced gas exchange performance through increased membrane surface density and shorter characteristic diffusion distances. TPMS‐based computational design algorithms have previously demonstrated the ability to redistribute flow pathways and reduce stagnation zones in architected structures [69]. Future integration of algorithm‐driven geometric optimization with high‐resolution additive manufacturing may ultimately enable patient‐specific artificial lung architectures tailored to individual anatomical and physiological conditions. Such developments would advance the realization of implantable and anatomically integrated biohybrid lung systems for long‐term respiratory support. Together, these findings support architected TPMS membranes as a promising framework for anatomically integrated biohybrid artificial lungs by combining membrane topology, additive manufacturing, and endothelialized biointerfaces within a unified design concept.

## Author Contributions

S.V.J., J.L. and B.W. conceptualized and designed the study. S.V.J. K.P.B. and U.S. performed the computational modeling and fluid dynamic simulations. M.P., C.N., G.S., F.N., L.T.H., S.B., C.C. and K.P.B. performed experiments, collected data, and conducted statistical analyses. M.P., C.N., and G.S. conducted biological and endothelialization experiments. J.L., F.N., L.T.H., S.B., and C.C. developed and fabricated the TPMS membrane structures and composite materials. S.V.J., K.P.B., U.S. and J.A. contributed to oxygenator engineering validation. J.A. provided expertise in organ support technologies and contributed to data interpretation. A.R. and B.W. supervised the translational and clinical aspects of the project. B.W., U.S., and M.W. were responsible for funding acquisition. S.V.J., M.P., J.L., F.N., L.T.H. and K.P.B. drafted the manuscript. U.S., M.W., J.A., A.R and B.W. critically revised the manuscript for important intellectual content. B.W. led the overall study, project administration, and serves as corresponding author. All authors approved the final version of the manuscript and agree to be accountable for all aspects of the work.

## Funding

This research was funded by Deutsche Forschungsgemeinschaft (DFG, Grant Nos: 347346497, 447746988, 347368182, and 286251789), Deutsches Zentrum für Lungenforschung (DZL, Grant No: DZL: 82DZL00201). The work was also supported by funds from the DFG's Gottfried Wilhelm Leibniz Prize 2019 (WE 4678/12‐1).

## Ethics

All procedures involving human‐derived materials were conducted in accordance with institutional guidelines and approved by the responsible ethics committee (No. 1303‐2012). Informed consent was obtained from all participants.

## Conflicts of Interest

The authors declare no competing financial interests. Matthias Wessling is a co‐inventor of a related pending patent (P. Bongartz, M. Meyer, M. Wessling, A. Scheele; “Method of additive manufacture of porous gas‐permeable shaped bodies having controllable porosity,” RWTH Aachen University, 2023, WO2021152128 A1). The patent is not directly related to the specific TPMS oxygenator design described in this study.

## Supporting information




**Supporting File**: adma74361‐sup‐0001‐SuppMat.docx.

## Data Availability

The data that support the findings of this study are available from the corresponding author upon reasonable request.
