# Supplementary material for: From Fiber Bundles to Architected Membranes: Triply Periodic Minimal Surface Architectures for Biohybrid Artificial Lungs
Source: Adv Mater. 2026 Aug 3;38(55):e74361. doi: 10.1002/adma.74361 (PMC13629250; doi:10.1002/adma.74361)
Supplement: Supplementary file 1 [file ADMA-38-e74361-s001.docx]

**From Fiber Bundles to Architected Membranes: Triply Periodic Minimal Surface Architectures for Biohybrid Artificial Lungs**

Michael Pflaum, Kai P. Barbian, Florian Neuhaus, Gerrit Sitarz, Carolin Nölke, Sebastian V. Jansen, John Linkhorst, Lukas T. Hirschwald, Sebastian Brosch, Christian Certa, Ulrich Steinseifer, Matthias Wessling, Jutta Arens, Arjang Ruhparwar, Bettina Wiegmann*

*** Corresponding author**

Prof. Dr. med. Bettina Wiegmann

Department of Cardiothoracic, Transplantation, and Vascular Surgery

Hannover Medical School

30625 Hannover, Germany

Email: wiegmann.bettina@mh-hannover.de

Supporting Information

*Table S1: Primer pairs used for gene expression analysis. All primer pairs were designed to work at 61°C annealing temperature.*

| Gene | Forward [5’-3’] | Reverse | Length [bp] | Gene access |
| --- | --- | --- | --- | --- |
| E-Selectin | ATC CAG CCA ATG GGT TCG TG | GAA GGC TCT GGG CTC CCA TT | 114 | NM_000450.2 |
| ICAM1 | CTA CCT CTG TCG GGC CAG GA | AGG CCT GCA GTG CCC ATT AT | 132 | NM_000201.2 |
| VCAM1 | GGC GCC TAT ACC ATC CGA AA | GAG CAC GAG AAG CTC AGG AGA A | 156 | NM_001078.3, NM_001199834.1, NM_080682.2 |
| Tissue Factor | CCC GAA CAG TTA ACC GGA AGA | GGA GTT CTC CTT CCA GCT CTG C | 191 | NM_001993.4 |
| Thrombomodulin | GCC CAT GGG AGC TGG TTA GA | GGC CTG ACT TGG CCT GCT AC | 190 | NM_000361.2 |
| ß-Actin | ATT GCC GAC AGG ATG CAG AA | GGG CCG GAC TCG TCA TAC TC | 176 | NM_001101.3 |

 *Figure S1: Morphological characterization of DLP-printed xPDMS membranes. Left: cross-sectional scanning electron microscopy (SEM) image demonstrating the dense, non-porous morphology of xPDMS. Middle: energy-dispersive X-ray (EDX) mapping confirming homogeneous silicon distribution throughout the membrane. Right: multilayer cross-sectional SEM image illustrating reproducible layer-by-layer fabrication with approximately 100 µm layer thickness per printed layer.*
